# Hospital Huts

**Published:** 1887-03-05

**Authors:** 


### Hut Hospitals.*

The proposal to substitute wooden huts as wards for buildings of a more lasting kind as a safeguard against the occurrence of septic diseases is no new one. Its origin dates from the observance during war times of the much higher percentage of recoveries from amputations and wounds of all kinds made in open sheds and mere temporary shelters than in buildings of brick or stone. Arguing from these data, it was asserted that wards ought in no case to be of a permanent nature, but should be formed of wood and periodically destroyed and replaced by others of a like kind. The advocates of this system overlooked, however, one very important element in the case, which is that the adoption of wooden structures means that ward pavilions must be limited to one storey in height. This, again, means that the hospital will cover more ground, with consequent increased cost for labour and supervision. The author of this pamphlet brings no new facts to bear in support of his system. He indeed goes further than the original advocates of huts, for he proposes to make them permanent, not temporary, buildings. As auxiliaries to the ordinary wards of a large hospital huts are undoubtedly of great value, but everything that can be said in favour of a hut applies with still greater force to a tent.

It is, however, not very easy to see the precise value of the system of periodically destroying the wooden wards. It is quite as possible, indeed, even more possible, to cleanse and disinfect a wooden hut, than to perform the same process on a brick building. Mr. English's proposal, therefore, to build huts as permanent structures, so formed that they can be readily taken to pieces and disinfected, is a reasonable one.

The form of ward devised by Mr. English is on plan a dodecagon, containing twelve beds. The sides are double, and the interspace between the outer and inner lining is filled in with charcoal or dry earth. Whether this last is a precaution of any value is open to question; possibly it might be better to leave the space free, or to utilise it in aid of the ventilation.

About the actual construction of the hut Mr. English does not say much; but if, as is to be inferred from the description, the sides and roof are all so made as to be easily removable, and capable of being readily transported from place to place, and withal to be weather-tight when erected, the hut should be of great value as a portable hospital in time of war.

* *Hints on Hut Hospitals*, by Edgar English, M.R.C.S. Stacy and Son, Kingsland, London, 1886.

